# In defence of medical judgement: medicalisation strategies in the daily life of the Lima Asylum in the last third of the 19th century

**DOI:** 10.1017/mdh.2025.10034

**Published:** 2026-04

**Authors:** Elias Amaya Nuñez

**Affiliations:** Universidad de Tarapaca, Chile

**Keywords:** Alienists, Asylum, Families, Medicalization, Psychiatric care, State agencies

## Abstract

This article analyzes the medicalisation strategies deployed by Peruvian alienists in the daily life of the Lima Asylum during the last third of the 19th century. Special attention is given to the process of hospitalisation of the insane in the psychiatric hospital, since this administrative procedure reveals the dialogue, confrontation, and negotiation between the asylum staff and the state and social bodies in the public management of insanity. Through the support of the civil authorities in charge of the psychiatric hospital administration, we argued that the local alienists sought to impose medical knowledge in the asylum space as the legitimate criterion for the confinement of the insane in Peru. This process was not without tensions, setbacks, and disputes, especially with the families and the state agencies of control and social defence seeking to preserve their former prerogatives over the fate of their insane. However, we propose that these medicalisation strategies promoted by the alienists in the daily space of the Lima Asylum managed to situate psychiatric care as a state problem and these actors as experts in the public management of insanity.

## Introduction

At the end of 1891, the alienist Manuel Antonio Muñiz informed the inspector of the Lima Charity Society that he was carrying out a radical reform in the organisation of the Lima Asylum, the only psychiatric hospital in Peru dedicated to public care for the mentally ill. This reform primarily focused on strengthening the alienist’s authority over the admission, care, and treatment of institutionalised patients, enforcing medical expertise over the intervention of other agents unconnected to alienism. One of the most significant changes Muñiz introduced was the creation of a mandatory special form required for patient admissions. This document demanded a more detailed record of data related to patients’ parentage, life history, and pathological background, aiming to explain their state of mental alienation. With the support of the authorities of the Charity Society, the form was distributed to families and various state offices across Peru, particularly to institutions responsible for defence, social control, and surveillance. To prevent potential arbitrariness by these groups, the asylum staff tightened control over the administrative admission process and required government institutions to comply with submitting the aforementioned documents. At the end of his annual report, Muñiz thanked the authorities of the Charity Society for complying with each of his directives. This formal gesture not only reflected the alienist’s gratitude but also acknowledged the network of non-medical actors and forms of knowledge that contributed to the operation of the Lima Asylum.

Since the early nineteenth century, the care of the insane in Latin America went through different ‘professionalising moments’, linked to both global and national processes that redefined the prevailing idea about madness.^1^ The transatlantic circulation of agents, knowledge, and technologies linked to European alienism – a reformist movement that, since the late eighteenth century, had promoted a process of medicalisation that defined madness as a distinct illness treatable by a specialist physician or alienist – enabled its medical proposals to be incorporated into modernisation projects of the young Latin American republics.^2^ It was common for medical elites to lead public campaigns in their respective countries to legitimise and institutionalise the practice, among which stood out the opening of special asylums – also known as lunatic asylums – where the insane could be isolated, monitored, and treated through moral therapy.^3^ These institutions were not only platforms for specialisation and professional training for physicians interested in the study of alienism, but also marked a turning point in the understanding, treatment, and public care of insanity. Although this knowledge lacked a sufficiently developed cognitive basis to offer a biological explanation of mental illness and an effective therapeutic treatment,^4^ this did not prevent alienists in the region from seeking institutionalisation as a medical discipline and state-sanctioned knowledge. Thus, starting from the second half of the nineteenth century, it is possible to identify various medical projects throughout Latin America focused on the medicalisation of the asylum system. A common feature of these initiatives was the defence of medical judgement against the intervention of other social actors and instances in the admission and discharge of patients.^5^

Thanks to the substantial revenues obtained in the mid-nineteenth century from guano exports – a natural fertiliser in high demand across European markets – the Peruvian state was able to implement various public policies aimed at strengthening its presence and control over social groups deemed problematic, including criminals, the sick, and the insane.^6^ The inauguration of the *Hospital Civil de la Misericordia* (also called Lima Asylum) in December 1859 formed part of these modernisation measures aimed at social order, control, and defence mechanisms. Although the psychiatric institution lacked the architectural grandeur of other infrastructure projects that were transforming Lima’s urban space and imposing new models of civility, its operation marked a turning point in transforming both the conditions and care for people with mental disorders in Peru.^7^ According to the foundational myth of Peruvian psychiatry, the opening of the Lima Asylum in 1859 ended the violent and inhumane treatment endured by mentally ill individuals in the archaic colonial-era madhouses of San Andrés and Santa Ana hospitals, institutions that persisted into the early republican period. As part of a regional trend embracing the therapeutic advances of European (primarily French) alienism, the asylum system allowed Peruvian alienists to reframe psychiatric patients as ‘sick’ individuals who, through a therapeutic regimen emphasising isolation, moralisation, and disciplined labour, could be ‘cured’ and reintegrated into society as productive subjects.^8^

The operation of the Lima Asylum fell far short of the expectations that had justified its establishment. During the second half of the nineteenth century, the psychiatric institution suffered the consequences of Peru’s turbulent political and socioeconomic context, which progressively limited its caregiving capacity and plunged it into gradual decline. The end of the guano boom, the 1868 yellow fever epidemic, the War of the Pacific against Chile (1879–1884), and the 1884–1885 civil war were among the events that directly impacted both the economy and infrastructure of the Lima Asylum.^9^ Moreover, the historical, political, and economic prominence of Peru’s capital, Lima, gradually transformed the asylum from a regional institution into a national one. As Peru’s sole state-run mental hospital, it established a monopoly on public mental healthcare, receiving not only patients from across the country but also the few existing specialists in mental medicine. These factors plunged the Lima Asylum into a progressive internal crisis due to overcrowding and limited economic resources, while simultaneously making it a key site for the institutionalisation and legitimisation of alienism. In the final third of the nineteenth century, Peru underwent a period of political stability and economic recovery that enabled significant advances in medical science: the consolidation of a national medical elite, the development of scientific research with international reach, and the design of state-led projects to modernise hospital infrastructure.^10^ In the realm of psychiatric care, alienist physicians pursued various professionalisation strategies aimed at both upgrading facilities and advancing the medicalisation of Lima Asylum.

Throughout the twentieth century, the historiography of Peruvian psychiatry, developed mainly by physicians, paid little attention to the study of the daily life of their most significant asylum institutions.^11^ The few works that went beyond the traditional historiographic approach and were influenced by an excessively critical perspective derived from Michel Foucault’s proposals described the alienist physician as an agent obsessed with social order and control, and the Lima Asylum as his repressive instrument.^12^ Recently, historian Andrés Ríos has brought us closer to the daily dynamics of the psychiatric hospital through the encounter, confrontation, and negotiation that the authorities and staff of the asylum had with the families and state bodies to define the admission of the insane.^13^ The study is based on his methodological proposal of a ‘social history of psychiatry’, but with a greater emphasis on the role of alienists as part of a medicalisation project.

In recent years, the historiography of psychiatry in Ibero-America has given us greater insight into the complex internal dynamics of asylum and psychiatric hospitals in the region and the fragile ‘psychiatric power’ of the doctors who worked in their facilities. These studies challenged the mechanical application of foreign interpretive schemes, especially those derived from the European ‘school of social control’ and raised the importance of local contexts in the institutional development of mental medicine.^14^ The development of a ‘history from below’ or a social history of psychiatry has made it possible to consider psychiatric institutions as a ‘social stage’ where diverse agents, knowledge, institutions, and devices that transcended the field of medical science converged.^15^ This resulted in the visibility of an extensive network of power relations composed of a plurality of agents (professionals, civil, and ‘profane’) that defined the daily dynamics of psychiatric institutions according to their current interests. Thus, alienist physicians were included in a network of forces where they interacted, confronted, and negotiated in defence of medical and institutional judgement with other actors with equal, greater, or lesser agency capacity.

This article aims to analyse the medicalising mechanisms and strategies that alienists deployed in the daily life of the Lima Asylum in defence of the medical and institutional competences questioned by the community and various state agencies. Particular attention is paid to the administrative process involved in the admission of the insane, as it draws attention towards those interactions, tensions, and confrontations generated between the psychiatric hospital staff and the social institutions. The time frame covers the last third of the nineteenth century. During this period, several and constant state and medical initiatives were taken to modernise the psychiatric care provided at the Lima Asylum. At the end of the nineteenth century, different coinciding events led to the paralysis of these reforms and a gradual decline of the psychiatric hospital – among the most significant are the premature death of alienist Manuel Antonio Muñiz and the preparations for the construction of a new national asylum.

This article is divided into three sections. The first one describes the organisation and hierarchical structure in charge of managing the Lima Asylum, with special emphasis on inspectors, nuns, and doctors. Thus, we also pay attention to the medical discourse deployed by the alienists against the organisation and administration of the psychiatric hospital. In the second section, we analyse the tensions and disagreements generated in the daily life of the asylum due to the failure of families and state agencies responsible for social order to comply with the admission requirements. Finally, the last section describes the mechanisms used by doctors and inspectors to defend the medical and institutional judgement against the transgression of the social actors mentioned above. Rather than emphasising the concrete results of this medical offensive, the purpose is to focus on the active role and strategies used by alienists to insert their medicalisation initiatives in an organisation and hierarchical structure that was hostile to them.

This examination of the daily operations at Lima Asylum has been made possible through the use of newly available documentary sources. Through the efforts of authorities and staff at the Museum of Psychiatric History of Lima’s Víctor Larco Herrera Hospital, in collaboration with the British Library’s Endangered Archives Programme (EAP 1402), a significant collection of administrative, financial, and medical records from the historic Lima Asylum has been preserved, catalogued, digitised, and made publicly accessible. This work draws on much of this material, especially the Copying books of notes and reports and correspondence of the Lima Asylum (1888–1899), as well as resources commonly used in historiographic narrative, such as medical journals, institutional memories, regulations, and medical reports. This documentary variety allows us to contrast the often-pessimistic medical discourse when it comes to analysing the reality of the asylum with its administrative documents.

## Organic composition of the Asylum and the alienists

Since its foundation in 1859, the daily dynamics of the Lima Asylum were determined by internal regulations that remained in force until 1897. These regulations specified the role, responsibility, hierarchy, salary, and routine of each of the members (inspectors, medical staff, nuns, accountants, and junior staff) that made the operation of the asylum possible. Rather than reflecting the reality of the institution *stricto sensu*, the regulations bring us closer to the vision held by authorities and state elites regarding what they considered to be most convenient for the public care of the insane.^16^ The institutional dynamics of psychiatric institutions in the region was often influenced and defined by external factors rather than by a strict medical classification and organisation. Thus, it is not surprising that the social processes and hierarchies that characterised the various modernisation projects conducted in the countries of the region have been reproduced in the asylum space. One of the most significant social processes has been the phenomenon of secularisation.^17^ The unique trajectory of this process in Latin American hospital care, which in some cases expelled religious elements while in others produced contentious coexistence, progressively shaped both the hierarchical structure and the roles of involved actors (civil, religious, and medical) within healthcare institutions.^18^ Our analysis will examine the responsibilities of the authorities and agents that directly influenced the administration of Lima Asylum. Particular attention will be given to: the professional standing of alienist physicians within the psychiatric hierarchy, and the development of a medicalising discourse aimed at challenging the roles played by other institutional stakeholders.

**Figure 1.**
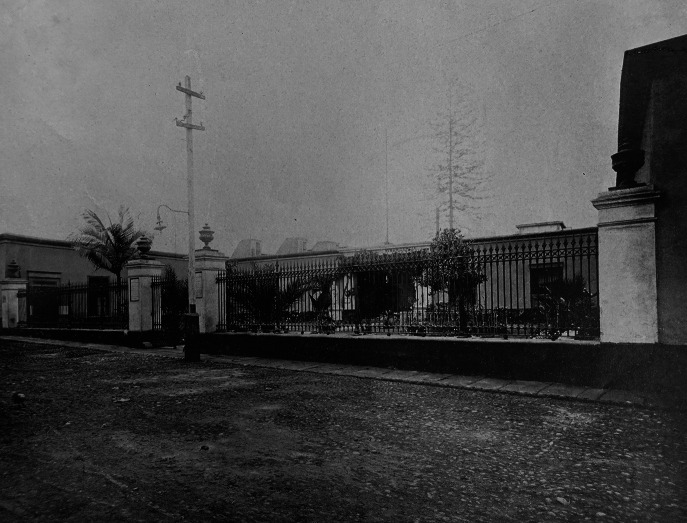
Main facade of the Hospital Civil de la Misericordia, also called Lima Asylum. Sources: Sociedad de Beneficencia Pública de Lima, *Álbum fotográfico* (Lima: Casa Editora M. Moral, 1913).

Since it was opened, the *Hospital Civil de la Misericordia* was considered a ‘public establishment’ destined to the ‘assistance and healing’ of the insane (Figure 1). As it belonged to the Lima Charity Society and even though its authority covered only the Peruvian capital, the asylum was part of a heterogeneous and extensive network of state and private institutions responsible for assisting, transferring, guarding, and confining the insane in the national territory. The director of the Charity’s General Board not only had authority over the psychiatric institution but also regulated the communication between the psychiatric institution and the state. However, with the administrative modernisation of government apparatus in the late nineteenth century, the Public Charity System was incorporated into a new state organisational structure, a process through which it gradually lost its autonomy as it became integrated into national healthcare and social policies.^19^ On the other hand, inside the asylum, representing the philanthropic institution, a partner held the position of ‘inspector’ or *mayordomo* (civil administrator). As the head of the asylum, the inspector had authority over all the activities conducted in its premises, and the employees (professional, religious, and civilian), as well as the patients, were ‘subject to his authority’.^20^ Since its foundation, the members of the Charity Society belonged to Peru’s elite class. By the late nineteenth century, as part of the country’s economic recovery, a segment of this rentier elite began shifting towards productive activities, embracing rational entrepreneurial attitudes.^21^ When appointed as inspectors, these members applied this pragmatic rationality at their discretion to their management, primarily focused on improving the psychiatric hospital’s financial administration. While this business-oriented approach frequently clashed with other stakeholders’ interests, particularly medical professionals’ criteria, its impact on the asylum’s modernisation processes during the final third of the nineteenth century remains undeniable.^22^

**Figure 2.**
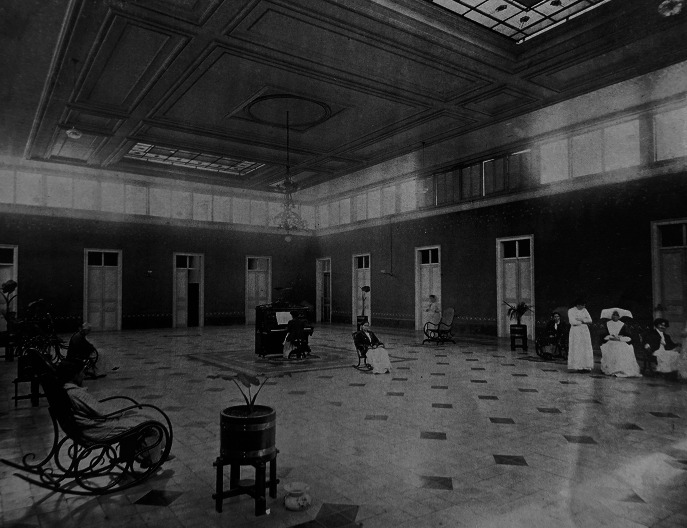
Female Pensioners’ Ward on the left side of the image are the Hermanas de la Caridad. Sources: Sociedad de Beneficencia Pública de Lima, *Álbum fotográfico* (Lima: Casa Editora M. Moral, 1913).

Although the regulations do not specify the hierarchical difference between the medical staff and the sisters of St. Vincent de Paul, in the dispositions of their articles, the nuns appear after the inspector and before the alienists. This nominal order that places nuns above doctors must be understood in a Latin American context that favoured the arrival of the European religious congregations actively involved in the administration and assistance of hospital, educational, and asylum institutions of the region.^23^ At the beginning of 1858, the first 45 Sisters of Charity arrived in Lima, taking over the administration of the main general hospitals of the capital.^24^ For the Medical Society of Lima, incorporating nuns into hospital care meant a reform ‘beneficial to science’ that would allow doctors to ‘rest’, since their prescriptions would be executed with the ‘severity demanded by science’.^25^ According to the regulations, the group of nuns was led by a mother superior who would organise other sisters in activities related to administration, order, assistance, cleaning, pharmacy, food, clothing, and all the details that would ensure the proper functioning of the asylum.^26^ During the second half of the nineteenth century, the number of nuns in the psychiatric hospital did not exceed six.^27^ However, this small number did not prevent the so-called *vicentinas* from maintaining control over the most important areas of the asylum, as they oversaw various employees responsible for carrying out their orders, such as guards, servants, laundresses, doormen, among others (Figure 2).

The medical service of the asylum was led by a ‘chief doctor’. He was entrusted with the direction of the psychiatric hospital regarding the ‘medical and sanitary’ aspect, considering the ‘moralisation’, healing, dietary regime, and any activity that involved the hygiene of the patients. Throughout the nineteenth century, Peru did not have official academic spaces to train specialists in the field of mental medicine. When the asylum was inaugurated, a single specialist was hired, as well as a medical student to accompany him. It was only in the last third of the nineteenth century that the number of medical staff increased to two regular doctors and two assistants. However, some doctors, as was the case throughout Latin America, found in transboundary and transnational travel, in the circulation of specialised literature and, above all, in the asylum, ways to delve into alienism. This was the case of the ‘founder’ of Peruvian psychiatry, alienist José Casimiro Ulloa (1829–1891), who in 1856 travelled to France to perfect his doctoral medical studies. During his stay in Europe, he learnt about the welfare reforms promoted by the first European alienism, through the visit to the ‘famous institutions for the insane’ of London and Paris.^28^ Upon his return to Lima, he encouraged a public campaign to put an end to the colonial madhouses and to found a new special asylum. Ulloa held the medical direction of the asylum until his death in 1891.

Although the local historiography has not paid attention to the pedagogical work performed by Ulloa in the asylum, there is no doubt that in its facilities, young students were trained by him during their annual professional internships. Manuel Antonio Muñiz (1861–1897), one of his ‘disciples’, had the greatest prominence as an alienist on the Peruvian medical scene. Since he started as an intern in the psychiatric hospital in 1884, Muñiz had an ascending professional career. Unlike his mentor, whose professional interests were directed towards the positioning of medicine as a professional field and as state-sanctioned knowledge, Muñiz turned his energies to medical specialisation, which showed in the defence of alienism and the renewal of psychiatric care in line with European and American medical contributions.^29^ Another student who, after his time as a medical intern, managed to delve into alienism and later became part of the medical staff was Leónidas Avendaño.^30^ Although some physicians outside psychiatry perceived the asylum as a transit institution until they could get better jobs, there were others, such as Wenceslao Salazar, who learned the speciality from close contact with alienists, even leading reforms in the field.^31^ Despite their small number, it is possible to identify in these alienists, through documentary sources, the birth of a differentiating identity in opposition to other collectives of physicians and laypeople who invaded the porous limits of mental medicine.

While everything seems to indicate that, in the daily life of the psychiatric hospital, the alienists adhered to internal regulations and rarely confronted their colleagues, yet their behaviour in the public sphere was quite different. Starting in the last third of the nineteenth century, like their counterparts in the region, Peruvian alienists articulated a medicalising discourse in which they positioned themselves as experts and as those responsible for the care of madness, directing their criticisms primarily at the institutional management of the asylum. Both Ulloa and Muñiz considered the psychiatric hospital as a therapeutic space where each element enabling its operation played a transcendental role in the moral recovery of the patient. These alienists conceived the asylum as an extensive and dense network enveloping the patients until they penetrated and regulated their thoughts, feelings, and movements.^32^ Given this special character of the institution, its direction could not fall to just any layman, since as Ulloa questioned himself, ‘Who but the physician, is the only competent person to give that direction in accordance with the precepts of science …?’^33^ In this way, the alienists claimed the ‘supreme authority’ through a position that would place them in the highest hierarchy of the asylum and that would combine administrative and medical activities. These interpretations and aspirations of the medical staff also show the permanence of the ideals of the early alienism in the Lima medical scene of the late nineteenth century: medical research was overshadowed by the public management of mental illnesses.

Even though most of the time they were cautious and diplomatic when questioning the administration and organisation of the asylum, there were certain situations in which the alienists directed their reflections towards the role played by their contemporaries within the institution. Although local historiography has emphasised the strong opposition that physicians showed to the work of the nuns, particularly highlighting the antagonistic nature of both groups (physicians–nuns, science–tradition, professional–empirical), the truth is that they equally questioned ‘non-medical’ subjects, often using gender and class stereotypes. According to Muñiz, it was a serious mistake that the most important position of the institution was held by a member of the Charity. Although the alienist took care not to question the moral integrity of these public men, he emphasised that the multiple private jobs they performed prevented them from conducting the supervision tasks and learning about the problems inside the asylum. As a result, the inspectors allowed the nuns to take on these functions ‘unrelated to their status and sex’.^34^ Although the intensity of opinions could vary, a faction of doctors agreed that the *vicentinas* were the ones who exercised the real authority in the asylum and in Lima’s hospital services. Unlike Ulloa, who was always cautious when referring to the performance of the Sisters of Charity, Muñiz was a convinced supporter of the lay service in the hospitals. The religious vows and those ‘precious qualities’, such as sacrifice, love, and charity, which at first had distinguished the *vicentinas*, had no value for the doctor, since in everyday life the sisters displayed the same passions, defects, and vices as ordinary people.^35^ Ulloa, for his part, subtly associated the administrative disorder within the asylum and the abuses committed against patients by the guards with the *vicentinas*’ lack of knowledge regarding scientific prescriptions.^36^ Nevertheless, both physicians recognised that, in the absence of qualified technical personnel, the complete elimination of religious service was not yet feasible. Rather than advocating for the outright expulsion of the sisters from the asylum – a goal that would become central to psychiatric reform efforts in the early twentieth century – the alienists aspired to circumscribe the nuns’ roles and place them under medical authority.^37^ Although these critiques emerged during moments of institutional crisis that brought the efficacy of asylum care into question, they should be understood within the broader historical process by which medicine was asserting itself within state structures and gaining legitimacy in Peruvian society.

## Tensions and disagreements in the daily life of the asylum: families and state institutions facing the admission of patients

While in the public sphere alienists openly rebuked their colleagues, the reality inside the Lima Asylum was a different story. The social history of psychiatry describes psychiatric institutions as a ‘social scenario’ whose inner dynamics were determined by a diversity of subjects, collectives, and institutions made up of specific knowledge often distant from medical science.^38^ In this way, the daily order of the asylum was shaped through a network of relations of interests that often manifested itself in dialogue, confrontation, or negotiation between the subjects and social instances involved.^39^ This historiographic perspective has not only broken the supposed hegemony of psychiatric power in the asylum space but also has positioned the agency of the alienist at the same level as other actors relegated by the historiography of social control, such as the patients themselves, the families, and the state agencies.^40^ One of the aspects that helps us approach this daily dynamic is the process of patient admission. As Andrés Ríos has rightly pointed out, admitting a sick person necessarily entailed an administrative process that would question the legitimate authority of the participating bodies to define the criteria for hospitalisation.^41^ As part of the asylum medicalisation process, the alienists defended their professional competence by exercising greater medical control over the admission of patients in the face of the intervention of the families and State authorities who had long claimed this power.^42^

As we will see in the following sections, although physicians were responsible for disseminating the admission procedures and emphasising the therapeutic value of asylum isolation, many families – and especially state agents tasked with defence and social control – failed to comply with these requirements. Instead, they often sought to override the medical and institutional authority of the asylum. In his study of the Peruvian prison system, historian Carlos Aguirre has shown that non-compliance with the internal regulations of Lima’s main prisons was a common practice, frequently tolerated by the very authorities in charge of their administration. The economic precariousness that hindered improvements to surveillance, control, and prisoner rehabilitation systems led higher-ranking officials to be permissive of infractions committed by subordinate staff and inmates, as such leniency helped to reduce internal conflicts and ensure the institution’s survival.^43^ As we will further explore, given the deep-rooted nature of this mode of exercising power within state institutions – of which the psychiatric hospital was no exception – it is not surprising that state agents transgressed the internal regulations of the Lima Asylum, thereby undermining medical authority.

In 1887, José Casimiro Ulloa stated that Peru lacked a law that would define the admission of the insane in the Lima Asylum. Instead, the psychiatric hospital’s internal regulations of 1859 had filled that gap.^44^ These regulations stipulated as an essential requirement for admission two medical certificates attesting to the mental condition of the patient. If it were an urgent admission of a case that would endanger public safety, the declaration of a priest or three neighbours would be enough. In the same circumstances, the ‘competent authorities’ could also refer the patients without an immediate requirement. On the other hand, the regulations also defined the categories of patients and the advantages they had in the asylum facilities. While poor patients in Lima received free care in common spaces, those who paid the first or second category board could access certain benefits depending on the amount provided, such as a private room, good food, and better recreation spaces.^45^ In the case of the alienated patients who were not from Lima or belonged to religious or state bodies, the families or the respective bodies that authorised their transfer and admission needed to make a monthly payment. In this way, the ‘free’ and ‘board-paying’ patients occupied the asylum facilities. Although the 1859 regulation remained in force until 1897, over the years the content of some articles, especially those on admission, release, and payment of boards, was modified according to current needs.

In the memoir mentioned before, Ulloa also differentiated between the crude imprisonment and the medical sequestration of the insane. In this way, he stated that since the insane person is a sick individual, their isolation in the asylum should not be considered as a violation of their individual freedom but rather as part of the medical treatment that they should receive. In order to protect the patient and social stability, Ulloa used to declare that alienists were the only specialists who should prescribe isolation and the place where the insane should be separated from the people and the environment that awakened their delirium.^46^ This authority should not be discretionary but rather should be regulated by the state. Ulloa called on the Lima Charity Society to create a commission of physicians and legal experts to discuss a bill on the assistance of the insane.^47^ The following year, in 1888, the inspector would promote some measures aimed at the disclosure of the requirements for the admission of patients and greater control when receiving medical certificates; however, there was no substantial change in the regulations.^48^ Why did the inspector choose to ratify the admission requirements rather than convening such commission? One possible answer can be found in the fragile respect that certain families and state entities had for admission criteria and board payments. Rather than a new regulation, it was necessary to enforce the already established stipulations.

With the aim of providing the asylum with financial resources, the inspectors promoted intense measures to collect board payment of the insane to the extent of visiting the homes of guardians and relatives, changing the status of ‘board-paying’ patients to ‘free’ patients, going to courts and, in the most extreme situations, trying to expel patients with debts. Although the mental asylum was being heavily criticised by some patients who claimed to have been victims of abuse and violence within its facilities, this did not stop a certain portion of the population from being interested in committing their ‘mad’ relatives there.^49^ The response of the families to achieve the admission and stay of their insane relatives in the psychiatric hospital was diverse, with the negotiation of debts, the use of bureaucratic strategies to avoid paying the board, and the timely abandonment of their family members standing out. It was common for physicians to report to the inspector that some families failed to deliver the medical records and certificates of their insane relatives. In 1890, Ulloa criticised the negligence of certain families in failing to provide the medical records of their insane relatives, since this was necessary to ‘form the most accurate judgment of their insanity’.^50^ Two years later, in 1892, Inspector Carlos Ferreyros would report this dramatic situation to the Charity: ‘Neither relatives nor representatives requesting the admission of an insane into the asylum provide the necessary information (…) once they obtain the admission of an insane, they forget about any subsequent obligation.’^51^ When the following year a form was issued requiring the recording of more detailed data on the sufferings of the insane,^52^ families continued to fail to comply with these measures.^53^

Even when families submitted the corresponding medical certificates, the alienists objected to them. For Muñiz it was a ‘scandal’ that in Peru a person was incapacitated with two medical certificates that did not explain the basis, symptoms, background, and causes of their derangement.^54^ Escaping the control of alienists, these certificates issued by colleagues from various specialities were perceived as an intrusion into a discipline such as mental medicine, which was struggling for legitimacy. Apparently, these ‘sheets of paper’ that did not provide any data about the disease^55^ made it possible for certain families to have their relatives admitted with relative ease and as many times as they deemed necessary whenever they regarded them as a problem within the domestic environment. In this way, it was quite common for relatives to ask for the admission, then the release, and, later, when any care became unsustainable, the re-admission of their insane.^56^ The following case exemplifies the above.

In July 1898, a member of a respected Lima family was admitted as a second-class patient due to his excessive alcohol consumption. Although his admission complied with the delivery of the medical certificates, Dr Wenceslao Mayorga observed that these came ‘completely blank’ with vague information referring to the ‘disorder’ of his intellectual faculties. After a period of observation and conversations with the patient, Mayorga was able to verify that he only had a temporary disorder as a result of drunkenness and that he had been hospitalised by his family to ‘correct’ him from the ‘vice’ of alcohol.^57^ The doctor requested his discharge, but to avoid any controversy about the patient’s hospitalisation, the inspector made it clear to the director of the Charity that the ‘honourability’ of the patient’s family could not be questioned.^58^ Months later, Inspector Domingo Olavegoya registered the re-admission of the alleged alcoholic, demanding that he should receive more careful attention.^59^ This time his behaviour would be different, since he would go from pleading for his freedom to threats and violence. This situation would reach its peak in April 1899, when Mayorga was assaulted by the patient, receiving several blows. On May 2, he was reported to be leaving the psychiatric hospital for ‘not being insane’.^60^ As Andrés Ríos has rightly pointed out, this and other cases show that, given the lack of power of the families in the domestic sphere, they used the psychiatric institution as a mechanism for control and punishment to correct their members, often contravening medical judgement or taking advantage of institutional regulations.^61^ But just as there were families who considered the asylum as a disciplinary option, there were others who perceived it as a therapeutic option and protection for their loved ones.^62^ In June 1899, anticipating her early death and the helplessness of her ‘idiot’ son, an 80-year-old widow asked the inspector to enter into an annuity agreement which would ensure the care of her offspring until the day of his death. Although this agreement was not at all profitable due to the good health of the patient and the small capital deposited, the application was accepted according to the charitable purposes of the Charity Society.^63^

Like many families, state agencies were also reluctant to comply with admission requirements. Although a variety of institutions were involved in shaping public care for the mentally ill, the following sections will focus on the role played by government bodies responsible for maintaining social order. As Andrés Ríos and Augusto Ruiz have pointed out, the police forces, as well as the families, were the agencies that referred the most insane to the asylum.^64^ The Lima police or civil guard held an important role in the documentary narrative of the psychiatric hospital, since due to their authoritarian nature and fragile institutionalisation, it was common for them to transgress the institutional and medical judgement regulating the customary order of the asylum. Although talking of a madmen hunt is an exaggeration,^65^ the action of the police forces in the admission of the insane must be understood within the framework of a repressive and violent policy aimed at punishing or preventing the behaviour of the lower classes that, according to the elite, threatened public order.^66^ This discretionary power of the police was revealed in the type and condition of the ‘insane’ who were sent to the asylum, as well as in the procedure for their admission. In 1883, Ulloa described some police members as possessed by ‘weak human feelings’, as they applied ‘cruel abuse’ to people picked up from the city streets, especially ‘furious’ subjects in a state of excitement. In this way, alleged insane people arrived at the psychiatric hospital often with bruises, serious injuries, or even in a state of agony.^67^

On the other hand, it was common for police officers to fail to present the required certificates or forms for admission, resorting instead to the pressure of force to secure the committal of patients.^68^ However, even when formal procedures were followed, many of these documents – like those submitted by families – lacked relevant information justifying the diagnosis. In this instance, the asylum authorities also directed complaints at the officials responsible for issuing such credentials. The so-called ‘police doctors’ acted as legal experts, issuing certificates and medical reports requested by prefecture authorities, police superintendents, and other state institutions.^69^ In 1894, Inspector Moscoso told the Charity director that police doctors refused to complete the information in the forms attached to the medical certificates and how this failure was detrimental to the work of alienists in preparing medical statistics.^70^ This warning was repeated in the following years and was spearheaded by physician Manuel Antonio Muñiz.^71^ The tension between alienists and police doctors becomes even more complex when considering that some doctors who worked at the asylum had previously been employed by the police. The most emblematic case is that of Dr David Matto, who, after Muñiz’s death, became director of the psychiatric hospital between 1899 and 1914, the year of his own passing.^72^ Although few managed to build careers as alienists and later as psychiatrists, these disputes must be understood within the framework of an institutionalisation process, in which both mental medicine and legal medicine vied to establish themselves as scientific disciplines and legitimate state-sanctioned knowledge.

The Ministry of Justice was another state agency that openly questioned the institutional competences of the asylum. The relationship between the authorities of the judiciary and the asylum was tense and often conflictive due to the type of patients who were sent there: offenders and criminals under investigation or convicted. The cases recorded in the Copying books of notes and reports and correspondence of the Lima Asylum evidence the authoritarianism with which the wardens, judges, prosecutors, and ministers acted to achieve the admission and stay of the criminally insane in the asylum. Everything suggests that, in the absence of a law to determine the legal fate of insane prisoners, the aforementioned officials saw the asylum as the ideal space to provide a legal, humane, and scientific response to the public problem of criminal insanity. This might be interpreted as progress and a legitimisation of alienism in the judicial field; however, the failure to meet admission requirements and the often-violent nature of the transfers reveal that judicial authorities were, in fact, seeking to evade any responsibility for the defendants. A clear example occurred in November 1892, when a military unit, under orders from the Minister of Justice, transferred four convicted prisoners from the Lima Penitentiary to the asylum without the required medical certificates. As expected, the nuns in charge of the admission process could do little against the authorities, allowing the entry of these prisoners who did not meet the basic requirements.^73^

Similarly, the prolonged stay of these inmates in the asylum, whether for medical treatment or mental health evaluation, caused significant discontent among staff, as it required resources the institution lacked. This was the case with an Asian man transferred to the asylum by court order in December 1897 for examination by the medical board. According to psychiatrist Wenceslao Mayorga, the lack of anthropometric equipment and the difficulty in finding a Chinese translator – specifically for ‘a dialect spoken by very few’ – delayed the completion of the medico-legal assessment.^74^ These delays incurred maintenance costs that later proved difficult to charge to the referring institutions due to the complexities of state bureaucracy. One such case occurred in September 1897, when a man accused of attempting to murder his father was sent to the asylum for a medico-legal evaluation. The delayed submission of judicial documents postponed the medical examination and extended the inmate’s stay until the end of that year. This situation triggered an unnecessary administrative dispute over which institution was responsible for covering his boarding costs.^75^

However, what concerned authorities the most was the potential danger these inmates posed to the safety of both patients and staff at the asylum. The institution lacked both properly trained personnel and adequate infrastructure to monitor, isolate, and control individuals who typically exhibited cunning, violent, unpredictable behaviour and defiance towards any authority figure. Contemporary psychiatrists suspected some might feign mental illness to first escape police custody and later avoid asylum supervision. A telling case occurred in December 1889 when the inspector refused to admit a defendant, notifying the Charity Society that the individual had not undergone prior evaluation by forensic physicians. With no conclusive evidence of mental alienation, the official denied admission, believing this to be an attempt to evade justice by feigning madness.^76^

The medical examinations conducted, and frequent escape attempts indicate that some inmates did not suffer from any mental illness but were rather exploiting the system to secure their freedom. In February 1899, at the request of the Ministry of Justice, psychiatrist Wenceslao Mayorga conducted a medico-legal evaluation that confirmed an asylum inmate described as ‘irritable and quarrelsome’ showed no signs of mental pathology. A similar case involved a man accused of attempted homicide who, when examined by Mayorga around the same period, was found to be fully mentally competent. Given their sound mental condition, authorities ordered their immediate transfer to Guadalupe Prison.^77^ From the authorities’ critical perspective, admitting judicially processed inmates not only jeopardised the asylum’s internal order but also subverted its therapeutic purpose, effectively converting it into a detention facility. This was precisely the concern raised by the inspector to the Charity director in May 1889 when attempting to admit two criminals. Faced with the inmates’ scandalous and immoral behaviour, the official maintained that the psychiatric institution, by its very medical nature, lacked both the necessary military infrastructure and personnel. To the inspector, the asylum should remain exclusively a space for treating the mentally ill, not for confining ‘hardened criminals’.^78^

## A medical counteroffensive?

**Figure 3.**
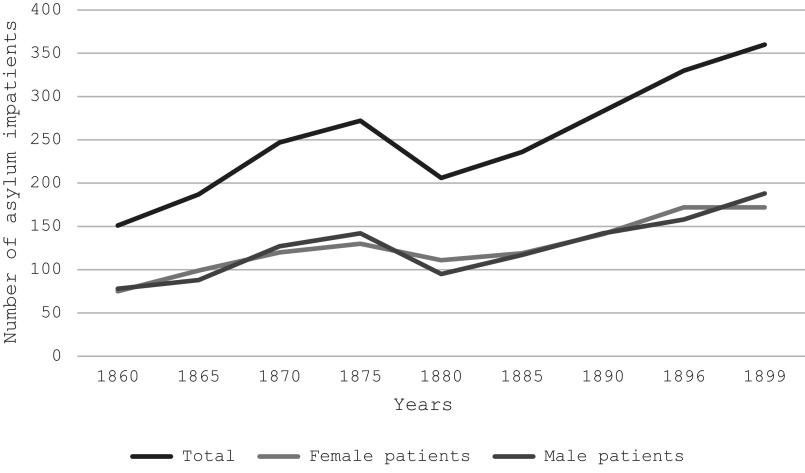
Patient population at the Lima Mental Asylum between 1860 and 1899. *Sources*: Muñiz, *op. cit.* (note 32), 126; Aspillaga, *op. cit.* (note 79), 83.

Since the founding of the asylum in 1859, the population of the asylum grew steadily, yet not exponentially. In 1860 the psychiatric hospital had 151 patients, both men and women; by 1899 the figure reached 360 (Figure 3).^79^ Although for Muñiz this increase was steady and accelerated, especially in the last third of the nineteenth century, it did not reach the alarming figures of the European and even Latin American countries.^80^ According to the alienist, although Peru was going through a political and socially convulsed stage that favoured the development of mental imbalances, the peculiarity of the Peruvian population, especially the ‘ethnic element’, and the geography could explain this ‘small number of insane people’. Since Peru was predominantly composed of an indigenous population that, owing to their Andean isolation and racial condition, was deemed ‘refractory to insanity’, the rise of mental illness was historically tied to the growth of industrial metropolises and urban living conditions.^81^ Consequently, psychiatrists routinely linked the development of certain mental pathologies to so-called ‘immoral’ or transgressive behaviours among specific racial and urban social groups, including poor whites, mestizos, Afro-descendants, and Chinese. Conditions like alcoholism and neurasthenia became particularly prevalent diagnoses, which medical professionals attributed to the disruptive effects of modern life on racialised populations such as immigrants, artisans, and the urban poor.^82^

Despite the low figures recorded by Muñiz, the growth of the asylum population had a direct impact on the institution’s infrastructure. Inaugurated in an old Jesuit building located in the Cercado neighbourhood, in the suburbs of the city of Lima, the internal layout of the asylum was subordinated to the conditions imposed by the original infrastructure. Thus, the original bipartite division of the building was used to create two large separate areas of men and women, and to divide each into five areas according to the illness that afflicted the patients and their status (quiet, calm, periodically excited, idiot, agitated, and furious insane). Although this distribution was directed by Ulloa, a few months after the opening of the psychiatric hospital, the alienist expressed his discomfort with the difficulty of isolating patients in each area.^83^ Over the years, questions about the infrastructure became more common and intense due to the increase in the number of patients, the material deterioration of the building, and the economic debacle of the Charity.

For the alienists, the overcrowding of their premises not only prevented therapeutic isolation, a fundamental precedent of moral treatment, but also caused the dangerous interaction between patients of different genders, races, and social classes. Although this contact enabled the spread of infectious diseases, what worried doctors most was the ‘contagion’ of the immoral customs of ‘free’ patients to distinguished paying ones.^84^ These criticisms reached their peak in the 1880s, when Ulloa and Muñiz conducted detailed studies on the organisation and infrastructure, concluding that in the psychiatric hospital, it was impossible to provide medical care in accordance with the advances of alienism.^85^ This time alienists were no longer just claiming supreme authority over the asylum, but were also demanding that the State authorities build a new psychiatric facility.

From the last third of the nineteenth century, the Lima Asylum underwent important administrative and material reforms, which would vary in intensity according to the management of each inspector. The priority of these authorities was to improve the economic situation of psychiatric hospital through measures to improve the collection, processing, and storage of patients’ personal information, especially the paying ones. However, these policies gradually covered different aspects of the administrative and material organisation of asylum and included the participation of other professional actors. The social recognition and professional prestige achieved in the dissemination and public practice of alienism by Ulloa and, especially, Muñiz helped to ensure that the recommendations of the medical body were considered in the internal policies promoted by the head of the asylum. In this way, although the interaction between inspectors and alienists was delimited by the institutional hierarchy, where often the judgement of the Charity was to the detriment of medical competences, alienists managed to promote certain measures in favour of the medicalisation of the asylum.

This process, in which the medical judgement sought to impose itself, was the result of intense negotiation, not without conflicts and setbacks, involving the most important state authorities, as well as the subordinate personnel in charge of implementing them daily. With the support of the Charity authorities, the medical staff expanded the number of its members, temporarily took control of religious personnel and guards, diversified medical services with novel therapeutic devices, participated in projects to expand the asylum areas and, particularly, coped with the intervention of other actors and entities outside the Lima Asylum. In keeping with the objectives of this article, we will focus on this last point, highlighting the manoeuvres that doctors and inspectors used to defend the medical and institutional judgement in the admission process of patients referred by the community, police forces, and provincial authorities.

As was the case in other parts of the world, the inspectors and doctors in Lima agreed on identifying the increase in the asylum population as one of the main problems affecting the psychiatric institution. In the critical view of these specialists, the overpopulation of the asylum not only had direct implications on medical care, as it made therapeutic isolation impossible, but also on the meagre economic budget, as it increased internal expenses. Although the 1859 regulations stipulated the admission requirements and the payment of boarding fees to patients with economic resources as well as those from outside Lima, these criteria became imprecise and outdated with the passage of time and the emergence of new legal categories of patients, like the ‘insane indigent’ or the ‘criminally insane’.

One of the early measures to regularise the admission process of the insane was focused on disclosing admission requirements and in recording more personal and medical information about patients. In February 1888, the Charity Inspector ordered to print 1,000 copies of a brochure containing some extracts from the rules of procedure relating to admission requirements.^86^ In the following months, a percentage of these copies was sent to the main hospitals of the city and to the ‘institutions referred to’ by the regulations.^87^ The reluctance of families and state agencies to comply with the requirements and the poor information on medical certificates prompted the medical staff to develop an additional form. In 1891, Muñiz stated that more than 80% of the patients who were admitted that year had brought a ‘defective medical certificate’ that did not contain any information about the diagnosis.^88^ Two years later, the alienist developed a ‘special form’ that required families and private practitioners to provide the personal and medical data of their insane.^89^ Although state authorities did little to comply with completing the files, given that in the following years there were many complaints from the doctors, the mere fact that these complaints were referred to the director of the Charity shows the inspector’s support for medical judgement.^90^

Parallel to the previous measure, the asylum authorities implemented other, more direct strategies aimed at formulating legal provisions that would specify certain articles of the regulations regarding the admission and the financial responsibility of the paying insane from outside Lima, as well as indigents, and criminals. For this purpose, the authorities of the psychiatric hospital sought the support of the director of the Lima Charity Society and, through the latter, of high governmental agencies. The relationship between the asylum staff and the authorities of the Charity and the state was complex, since each entity sought to impose its own interests and institutional judgement, often to the detriment of medical competence. This became evident in the case of the paying insane. It had become common practice for families to make monthly payments below the established amounts. Charity officials were increasingly involved in regulating the collection of boarding payments: They initially determined the exact amounts to be paid, established legal contracts with the guardians and, finally, awarded themselves the administration and collection of patients’ payments.^91^ It is evident that the regulation of the income from paying patients in favour of the interests of the Charity had an economic background, since this was not reflected in the requirements to release them. On the contrary, the authorities did very little to control the interference of the families when requesting the release of their insane. Although the alienists and the inspectors requested to prohibit the release of the insane without medical authorisation, these requests did not have any results.^92^ In 1897, the new regulations for the asylum would reaffirm the power of families and guardians to discharge the patients according to their priorities.^93^

Although institutional hierarchies had an important weight in policies regarding the public care of insanity, doctors managed to influence some of these decisions. Even though the asylum was designed to house the insane of Lima, the lack of similar institutions in the Peruvian territory meant that it gradually became a national dependency, even against the authorities of the asylum and the Lima Charity Society.^94^ Since the late nineteenth century, significant tension has existed between the authorities in Lima and those in the rest of Peru. The insane referred by the latter group never reached exorbitant numbers, yet they became problematic when they were abandoned by their relatives in Lima, only to be located and institutionalised by the police forces in the asylum. In the absence of legal guardianship, these individuals were classified as ‘indigent insane’, a designation that compelled the Charity Society to bear the costs of their care.^95^

As seen earlier, the alienists publicly and repeatedly questioned the poor rigour of police medical certificates and the unfortunate physical condition in which indigent insane arrived at the psychiatric institution. To stop these transfers, the asylum and the Charity authorities decided to accept government support. From 1875 to 1888, three supreme resolutions were issued prohibiting the authorities of non-Lima provinces from sending their patients due to the lack of space in the asylum facilities.^96^ It was only in August 1896, in response to the complaint of inspectors and doctors, that the government issued a new supreme resolution, but this time establishing that the transfer of indigent insane from outside Lima by the police needed to have the approval of the provincial authorities of the jurisdiction to which the patient belonged. These officers were also responsible for issuing medical certificates and forms and for complying with boarding payments.^97^ Although the asylum staff used the law to enforce admission requirements, when it was not enough, they used other resources to combat police authoritarianism. The doctors began to discharge the insane referred by the law enforcement agencies. In 1898, the release of indigent insane was so frequent that the police expressed their discomfort to the Charity. Doctor Eduardo Sánchez Concha explained that this was because the patients suffered from a temporary ‘toxic madness’ due to the drunkenness, and that the attentive care they received enabled their quick release.^98^ Both doctors and inspectors agreed that the asylum was not a correctional house to detain alcoholics in the city, but rather a therapeutic space governed by the prescriptions of mental medicine.^99^

## Conclusions

In the last third of the nineteenth century, several ‘professionalising moments’ can be identified in Latin America; their purpose was to institutionalise and legitimise alienism. The special importance of the asylum for nineteenth-century alienism, a therapeutic space that met a series of conditions for the deployment of medical power, made it the main stage of the various medicalisation projects promoted in the region. The organisation of these institutions often replicated the hierarchy and social processes experienced by Latin American societies since the mid-nineteenth century. In the Peruvian case, from its opening in 1859 until its closure in 1918, the Lima Asylum maintained an organic structure in which the governing power was structured by a group of agents, including civilians, religious, and professionals, with alienists occupying a secondary position. At the same time, as the psychiatric unit belonged to the Lima Charity Society, it was subject to a wider state network covering the entire national territory and to a heterodox group of public and private bodies. The lack of a clear definition of the roles and responsibilities of these agents and bodies in the public care of insanity, because of a state that was beginning to define its powers in public health, caused tensions and conflicts with the Lima Asylum personnel in defining the admission criteria of the community’s insane and of the social defence bodies.

Families, police forces, members of the judiciary, provincial authorities, among others, did little to meet the admission requirements and the payment for the insane board, and when they encountered obstacles, they did not hesitate to use force and violence. This intransigence not only distorted the therapeutic purpose of the institution, as there were patients who were admitted as a means of punishment, or simply abandoned, but also had a direct impact on the infrastructure and economy of the institution. Thus, the medical and institutional narrative for explaining the decline of the asylum at the end of the nineteenth century linked the limited control in the admission of patients with overpopulation, overcrowding, and the economic debacle of the psychiatric institution. Because of their own training, many of them belonging to the renewed national economic elite, the inspectors conceived the problems of the asylum in economic terms and thus promoted a series of administrative reforms to improve internal revenues.

The prestige that doctors such as Casimiro Ulloa and Manuel Antonio Muñiz had achieved through an active presence in public opinion and state bodies as experts in the field of mental pathologies helped to incorporate their medicalising projects – although often with amendments – in the administrative policies of inspectors. Since the 1890s, doctors have used different mechanisms to strengthen and impose medical and institutional judgement in the admission and discharge of patients arriving at psychiatric facilities. The creation of new forms to collect further information on patients, the review and questioning of medical certificates issued by non-specialists, and the constant demand to the authorities to prohibit the admission of patients referred by the police and judicial authorities were some of the measures applied by the medical body and the inspectors.

Although this medical counteroffensive did not always have the expected outcomes, since the state authorities continued to fail to meet the admission requirements or tried to influence the laws enacted according to their interests, it did have an impact on the public management of insanity. Alienists positioned the problem of insanity as a state problem, while placing themselves as the only experts in its identification, solution, and management. The laws enacted to regularise the admission of patients to provincial, judicial, and police centres were accompanied by other measures in which government authorities became directly involved in the management of insanity. In March 1896, the Minister of Public Works pointed out that the insane had a ‘right’ to the care of society and that this was a ‘pressing duty of the authority’ that could not be ignored by civilised people.^100^ Three months later, the government invited doctors to participate in a public tender to choose the best study for the construction of a new asylum facility based on the progress made by medical science.^101^

## Footnotes

1

Claudia Araya , La locura es nuestra, Profesionalización de la psiquiatría en Chile. Saberes y prácticas (1826–1949) (Santiago: Prohistoria, 2018), 16–35 .

2

Rafael Huertas , La Locura (Madrid: CSIC and Los libros de La Catarata, 2014), 67–92 .

3

María José Correa , ‘De la Casa de Orates al juzgado: pericia alienista y evaluación judicial de la locura en Santiago de Chile hacia 1860’, História, Ciências, Saúde-Manguinhos 20, 2(2013), 571–585 23903917 10.1590/s0104-59702013000200012.

4

Rafael Huertas , Organizar y persuadir. Estrategias profesionales y retóricas de legitimación de la medicina mental española (1875–1936) (Madrid: Frenia, 2002), 15, 33 .

5

Cristina Sacristán , ‘La medicalización de la locura en la Ciudad de México. De la secularización de los hospitales para dementes al cierre del Manicomio General, 1861–1968’, in Andrés Ríos and Marino Ruperthuz (eds.), De manicomios a instituciones psiquiátricas (Ciudad de México: Silex, 2022), 275–316, 285–286; Cristina Sacristán , ‘¿Quién me metió en el manicomio? El internamiento de enfermos mentales en México, siglos XIX y XX’, Relaciones 19, 74 (1999), 203–232 .

6

Carlos Aguirre , Dénle duro que no siente. Poder y trasgresión en el Perú Republicano (Lima: Fondo Editorial Pedagógico San Marcos, 2011), 93–113 ; Augusto Ruiz , Psiquiatras y locos. Entre la modernización contra los Andes y el nuevo proyecto de modernidad. Perú: 1850-1930 (Lima: Instituto Pasado & Presente, 1994), 24–27 .

7

Natalia Majluf , Escultura y espacio público. Lima 1850–1879 (Lima: IEP, 1994); Oscar Valdivia , ‘La psiquiatría en la época republicana’. Revista del Cuerpo Médico 2, 5 (1963), 5–81, 5.

8 Ruiz *op. cit.* (note 6), 31–36.

9

Carlos Contreras and Marina Zuloaga , Historia mínima del Perú (Lima: El Colegio de México and Turner, 2016), 181–211 .

10

Marcos Cueto , Excelencia científica en la periferia (Lima: Grade-Concytec, 1989).

11 See Santiago Stucchi , Loquerías, manicomio y hospitales psiquiátricos de Lima (Lima: Universidad Cayetano Heredia, 2012); Oscar Valdivia , Panorama de la psiquiatría en el Perú (Lima: UNMSM, 1989); Baltazar Caravedo , La reforma psiquiátrica en el Perú (Lima: Clínica Baltazar Caravedo, 1985).

12 Ruiz *op. cit.* (note 6); Augusto Ruiz , ‘Medicina mental y modernización: Lima, 1850–1900’, in Aldo Panfichi y Felipe Portocarrero (eds), Mundos interiores: Lima 1850–1950 (Lima: Universidad del Pacífico), 371–396.

13

Andrés Ríos , Locura y psiquiatría en Perú (1859–1947). Instituciones, miradas, juicios y prejuicios (Lima: UNMSM-UNAM, 2023), 27–61 .

14

Rafael Huertas , ‘Historia de la psiquiatría ¿por qué? ¿para qué? Tradiciones historiográficas y nuevas tendencias’, Frenia 1, 1 (2001), 9–36 ; Cristina Sacristán , ‘La locura se topa con el manicomio. Una historia por contar’, Cuicuilco 16, 45 (2009), 163–189 .

15

Andrés Ríos and Marino Ruperthuz (eds.), De manicomios a instituciones psiquiátricas (Ciudad de México: Silex, 2022), 16 .

16

Cristina Rivera-Garza , ‘Por la salud mental de la nación: vida cotidiana y Estado en el Manicomio General de La Castañeda, México 1910–1930’, Secuencia, 51 (2001), 56–89, 6219711556.

17

Cristina Sacristán , ‘Historiografía de la locura y de la psiquiatría en México De la Hagiografía a la historia posmoderna’, Frenia 5, 1(2005), 9–33, 26.

18 Ríos and Ruperthuz, *op. cit.* (note 15), 17–19.

19

Leticia Quiñones , Construir y modernizar: el Ministerio de fomento (1896–1930) (Lima: EDUNI, 2014), 21 .

20 Sección de Beneficencia, ‘Proyecto de Reglamento para el Hospicio de Insanos’, *El Peruano*, 11 April 1860, 107–109.

21

Felipe Portocarrero , Grandes fortunas en el Perú: 1916–1960 (Lima: Universidad del Pacífico, 2013), 64–65 .

22 Museum of Psychiatric History of Lima’s Víctor Larco Herrera Hospital, Copying books of notes and reports and correspondence of the Lima Asylum (hereinafter CBNRCLA), 22 March 1897, 384; 30 March 1897, 385–386.

23

Sol Serrano , Vírgenes viajeras. Diarios de religiosas francesas en su ruta a Chile, 1837–1874 (Santiago, Ediciones Universidad Católica de Chile, 2000), 13–112 .

24

Oscar Pamo , La medicina francesa en el Perú del siglo XIX (Lima: Perú Offset Editores, 2016), 61–69 .

25

Crónica Médica , Gaceta Médica de Lima 2, 35(1858), 131 .

26 Sección de Beneficencia, ‘Proyecto de Reglamento para el Hospicio de Insanos’, *El Peruano*, 11 April 1860, 107–109.

27

Carlos Ferreyros , Memoria que presenta a la Sociedad de Beneficencia Pública de Lima su director Sr. D. Carlos Ferreyros correspondiente al año económico de 1896 (Lima: Imprenta Liberal, 1896), 35 ; Francisco Carassa , Memoria de la Sociedad de Beneficencia Pública presentada por su director Don Francisco Carassa a la Junta General reunida el 28 de diciembre de 1870 (Lima: Tipografía de L. J. Tola, 1871), 28 .

28

José Casimiro Ulloa , Crónica interior. Loquerías, Gaceta Médica de Lima 2, 35 (1858), 131 .

29

Manuel Antonio Muñiz , ‘Memoria de los trabajos del año académico por el Secretario Perpetuo Dr Manuel A. Muñiz’, El Monitor Médico 12, 247 (1897), 159–169 .

30

Hermilio Valdizán , Diccionario de Medicina Peruana. Tomo II (Lima: Talleres Gráficos de Hospital Víctor Larco Herrera, 1938), 102 .

31

Carlos Ferreyros , Memoria que presenta a la Sociedad de Beneficencia Pública de Lima su director Sr. D. Carlos Ferreyros correspondiente al año económico de 1895 (Lima: Imprenta Liberal, 1895), 167 .

32

Manuel Antonio Muñiz , Asistencia pública de los enajenados (Lima: Imprenta La Industria, 1897), 124 ; Felipe Varela , Memoria presentada a la Sociedad de Beneficencia Pública de Lima por su director el Dr. Felipe Varela y Valle a la Junta General reunida el 31 de diciembre de 1883 (Lima: Imprenta Teatro, 1884), 68 ; José Casimiro Ulloa , ‘Informe’, El Monitor Médico 2, 12 (1886), 183–185, 183.

33 Varela, *op. cit.* (note 32), 67.

34

Manuel Antonio Muñiz , ‘Manicomio de Lima. 1884’, La Crónica Médica 2, 15 (1885), 93–95, 94.

35
*Ibid.*, 94; Octavio Tudela , Memoria que presenta a la Sociedad de Beneficencia Pública de Lima su director Dr. D. Octavio Tudela correspondiente al año económico de 1893 (Lima: Imprenta Torres Aguirre, 1894), 135 .

36 Varela, *op. cit.* (note 32), 67.

37 Muñiz, *op. cit.* (note 32), 67.

38 Ríos and Ruperthuz, *op. cit.* (note 15), 16.

39 Ríos, *op. cit.* (note 13), 31, 45.

40 Sacristán, *op. cit.* (note 5), 285–286.

41 Ríos, *op. cit.* (note 13), 45.

42 Sacristán, *op. cit.* (note 5), 285–286.

43

Carlos Aguirre , Donde se amansan los guapos. Las cárceles de Lima, 1850–1935 (Lima: Fondo Editorial Universidad del Pacífico, 2019), 201–215 .

44

Pedro Correa , Memoria presentada a la Sociedad de Beneficencia Pública de Lima pro su director el Sr. D. Pedro Correa y Santiago en la Junta General el 19 de diciembre de 1887 (Lima: Imprenta Calle del Cuzco, 1888), 36 .

45 Sección de Beneficencia, ‘Proyecto de Reglamento para el Hospicio de Insanos’, *El Peruano*, 11 April 1860, 107–109.

46 Correa, *op. cit.* (note 44), 40.

47
*Ibid.*, 41.

48 CBNRCLA, 24 February, 1888, 20.

49 Ruiz *op. cit.* (note 6), 53–71.

50

Manuel Candamo , Memoria presentada a la Sociedad de Beneficencia Pública de Lima por su director Sr. D. Manuel Candamo en la Junta General reunida el 23 de diciembre de 1891 (Lima: Imprenta y librerías de Benito Gil, 1892), 80 .

51 CBNRCLA, 6 December 1892, 284–289.

52 CBNRCLA, 20 December 1893, 320.

53 CBNRCLA, 8 February 1899, 532; 23 February 1889, 536; 25 February 1899, 538; 20 May 1899, 559.

54 Tudela, *op. cit.* (note 35), 138.

55
*Ibid.*, 138. CBNRCLA, 26 November 1898, 510.

56 CBNRCLA, 31 July 1891, 236; 26 May 1898, 469–470; 28 May 1898, 470; 4 October 1897, 419.

57 CBNRCLA, 18 July 1898, 482–484.

58 CBNRCLA, 22 July 1898, 485–486.

59 CBNRCLA, 2 November 1898, 503–504.

60 CBNRCLA, 2 May 1899, 555.

61 Ríos, *op. cit.* (note 13), 49.

62 Sacristán, *op. cit.* (note 5), 291.

63 CBNRCLA, 17 June 1899, 568–569.

64 Ríos, *op. cit.* (note 13), 31, 45; Ruiz, *op. cit.* (note 6), 46.

65 Ruiz, *op. cit.* (note 6), 44–45.

66 Aguirre, *op. cit.* (note 43), 112–121.

67 Varela, *op. cit.* (note 32), 65. CBNRCLA, 14 December 1888, 112–113.

68 CBNRCLA, 25 October 1895, 361.

69

Manuel Olaechea y Rufino López , ‘Medicina legal. Organización del servicio médico de policía’, La Gaceta Médica 5, 11 (1879), 324–327 .

70 CBNRCLA, 4 May 1894, 323.

71 CBNRCLA, March 1894, 328; 26 November 1898, 510.

72 ‘Médico de policía de Lima’, *La Crónica Médica* 6, 61 (1889), 5.

73 CBNRCLA, 9 November 1892, 281–282.

74 CBNRCLA, 24 December 1897, 443–444.

75 CBNRCLA, 25 September 1897, 417; 13 November 1897, 427–428; 29 November 1897, 431; 21 December 1897, 442–443.

76 CBNRCLA, 11 December 1889, 179.

77 CBNRCLA, 11 December 1889, 179.

78 CBNRCLA, 16 May 1889, 139–140.

79 Muñiz, *op. cit.* (note 32), 126; Antero Aspillaga , Memoria administrativa. Sociedad de Beneficencia Pública de Lima de su director Sr. D. Antero Aspillaga correspondiente al año 1899 (Lima: Torres Aguirre, 1899).

80 Ríos, *op. cit.* (note 13), 35.

81 Muñiz, *op. cit.* (note 32), 52–57.

82 Ruiz *op. cit.* (note 6), 46–47; Paulo Drinot , ‘Locura, neurastenia y modernidad. Interpretaciones medicolegales y populares del suicidio en la República Aristocrática’, in Marcos Cueto , Jorge Lossio and Carol Pasco (eds.), El rastro de la salud en el Perú (Lima: IEP and Universidad Peruana Cayetano Heredia, 2009), 211–258, 229–237.

83 Caravedo, *op. cit.* (note 11), 39–40.

84 Tudela, *op. cit.* (note 35), 134, 164. Ferreyros, *op. cit.* (note 31), 168.

85 Muñiz, *op. cit.* (note 34), 53; Varela, *op. cit.* (note 32), 64–66.

86 CBNRCLA, 24 February 24 1888, 20.

87 CBNRCLA, 1 March 1888, 24; July 4, 1888.

88 Candamo, *op. cit.* (note 50), 115.

89 Tudela, *op. cit.* (note 35), 137–138.

90 CBNRCLA, 11 June 1894, 335.

91 CBNRCLA, 3 February 1888, 4–5; 10 July 1888, 54; 11 October 1888, 91; February 1894, 326; 24 April 1897, 387–388.

92 CBNRCLA, 25 August 1888, 78–79; 16 May 1896, 378; 4 October 1897, 419.

93
*Reglamento provisional del Hospicio de Insanos* (Lima: Imprenta Liberal, 1897), 22.

94

Ramón Azcárate , Memoria de la Sociedad de Beneficencia Pública presentada por el segundo vice-director encargado de la dirección Señor Don Ramón Azcárate a la Junta General, reunida el 29 de diciembre de 1879 (Lima: Imprenta de la Merced, 1880), 45 .

95 CBNRCLA, 7 December 1891, 247–250.

96 CBNRCLA, 11 October 1888, 34; 20 October 1888, 93–96; 7 July 1891, 235.

97

Sociedad de Beneficencia Pública de Lima, Reglamento orgánico. Leyes, resoluciones supremas, acuerdos y otros documentos pertinentes (Lima: Impr. El Bien Social, 1907), 90–91 .

98 CBNRCLA, 24 June 1898, 474.

99 CBNRCLA, 25 June 1898, 476; 22 July 1898, 485–486.

100

Ministerio de Fomento, Registro oficial de Fomento. Minas, industria, beneficencia (Lima: Imprenta de la Escuela de Ingenieros, 1896), 45–46 .

101

Elías Amaya , ‘Ingenieros, arquitectos, médicos y locura La construcción del Asilo Colonia de la Magdalena (1896–1918)’, Devenir 5, 10 (2018), 65–90. .

